# The Achilles tendon total rupture score: a study of responsiveness, internal consistency and convergent validity on patients with acute Achilles tendon ruptures

**DOI:** 10.1186/1477-7525-10-24

**Published:** 2012-02-29

**Authors:** Rebecca S Kearney, Juul Achten, Sarah E Lamb, Nicholas Parsons, Matthew L Costa

**Affiliations:** 1 Warwick Orthopaedics, Division of Health Sciences, Warwick Medical School, Clinical Sciences Research Laboratories, University Hospital, Clifford Bridge Road, Coventry CV2 2DX, UK; 2 Warwick Clinical Trials Unit, Division of Health Sciences, Warwick Medical School, Clinical Trials Unit, The University of Warwick, Coventry CV4 7AL, UK

**Keywords:** Achilles tendon, Rupture, Questionnaires, Treatment Outcome

## Abstract

**Background:**

The Achilles tendon Total Rupture Score was developed by a research group in 2007 in response to the need for a patient reported outcome measure for this patient population. Beyond this original development paper, no further validation studies have been published.

Consequently the purpose of this study was to evaluate internal consistency, convergent validity and responsiveness of this newly developed patient reported outcome measure within patients who have sustained an isolated acute Achilles tendon rupture.

**Methods:**

Sixty-four eligible patients with an acute rupture of their Achilles tendon completed the Achilles tendon Total Rupture Score alongside two further patient reported outcome measures (Disability Rating Index and EQ 5D). These were completed at baseline, six weeks, three months, six months and nine months post injury. The Achilles tendon Total Rupture Score was evaluated for internal consistency, using Cronbach's alpha, convergent validity, through correlation analysis and responsiveness, by analysing floor and ceiling effects and calculating its relative efficiency in comparison to the Disability Rating Index and EQ 5D scores.

**Results:**

The Achilles tendon Total Rupture Score demonstrated high internal consistency (Cronbachs alpha > 0.8) and correlated significantly (p < 0.001) with the Disability Rating Index at five time points (pre-injury, six weeks, three, six and nine months) with correlation coefficients between -0.5 and -0.9. However, the confidence intervals were wide. Furthermore, the ability of the new score to detect clinically important changes over time (responsiveness) was shown to be greater than the Disability Rating Index and EQ 5D.

**Conclusions:**

A universally accepted outcome measure is imperative to allow comparisons to be made across practice. This is the first study to evaluate aspects of validity of this newly developed outcome measure, outside of the developing centre. The ATRS demonstrated high internal consistency and responsiveness, with limited convergent validity. This research provides further support for the use of this outcome measure, however further research is required to advocate its universal use in patients with acute Achilles tendon ruptures. Such areas include inter-rater reliability and research to determine the minimally clinically important difference between scores.

All authors have read and concur with the content of this manuscript. The material presented has not been and will not be submitted for publication elsewhere, except as an abstract. All authors have made substantial contributions to all of the following: (1) the conception and design of the study, or acquisition of data, or analysis and interpretation of data, (2) drafting the article or revising it critically for important intellectual content and (3) final approval of the submitted version. This research has been funded by Arthritis Research UK, no conflicts of interests have been declared by the authors.

Kind Regards

Rebecca Kearney (corresponding author)

Research Physiotherapist

## Background

Appropriate patient reported outcome measures (PROMs) are imperative for the evaluation of clinical practice and research [1]. This is exemplified by the increasing use of such measures during the last two decades. Examples of widely used measures within musculoskeletal care include the Victorian Institute of Sports Assessment questionnaire for patella and achilles tendinopathy [2,3] (VISA and VISA A questionnaires), the oxford hip and knee scores [4,5], and the Disabilities of the Arm, Shoulder and Hand score (DASH)[6].

A range of PROMs have also been published specifically for evaluating patients following a rupture of their Achilles tendon. Examples of these include the American Orthopaedic Foot and Ankle Hindfoot score [7], Thermann score [8] and Leppilahti score [9]. However the development of these measures have predominantly been based on expert opinion, with a lack of data to support their validation [10]. In 2007 a research group addressed this gap through development of a new patient reported outcome measure, with supporting validation data [10]. This outcome measure was the 'Achilles tendon Total Rupture Score' (ATRS).

The ATRS contains ten items, for which patients are asked to respond using an 11-grade Likert scale, by ticking one box labelled 0-10. This score of zerois equivalent to a patient having major limitations/symptoms and a score of ten is equivalent to a patient having no limitations or symptoms. The score was originally developed and evaluated in Swedish, using a sample of patients aged 20-70 years with an acute Achilles tendon rupture. The score evaluates the constructs of *'symptoms and physical activity*' through five questions addressing symptoms and five questions addressing physical activity.

The purpose of this study was to evaluate internal consistency convergent validity and responsiveness of the ATRS within a UK population of patients who had sustained an isolated acute Achilles tendon rupture.

## Methods

### Population identification

Ethical approval for this study was gained. Between August 2007 and June 2009, 70 patients presenting at the University hospital with an isolated, acute (< 10 days) midsubstance rupture of the Achilles tendon were screened. The diagnosis was established through subjective history and physical examination to confirm a palpable gap and a positive Thompson test [11].

Patients with delayed presentation, bilateral ruptures or other serious lower limb injury were excluded. In addition, patients who were unable to read the English language and therefore unable to complete the questionnaires were also excluded. Of the 70 screened patients, six were excluded. Three were excluded because they were unable to complete the questionnaires and three declined to complete the questionnaires.

Following non-operative or operative repair of their Achilles tendon, all patients were managed using an immediate weight-bearing protocol. This consisted of wearing an ankle foot orthoses with inserted heel wedges for eight weeks, followed by a standardised physical therapy programme.

### Questionnaire follow up

All patients were routinely evaluated at baseline, six weeks, three months, six months and nine months, using PROMs, which included the ATRS, EQ 5D and Disability Rating Index (DRI).

. In contrast to the ATRS, the DRI is not disease specific and has been validated for use in a range of orthopaedic presentations [12]. It is a self administered questionnaire with twelve questions regarding common physical activities, to which patients respond using a 100 mm visual analogue scale. There are two anchor points, 'without difficulty = 0' and 'not at all = 100'. The EQ 5D was also completed by patients to obtain a global measure of health [13]. The EQ 5D comprises of five questions which ask the patient to respond with one of three options. The combination of these responses results in a single health index score.

These scores were selected instead of the VISA-A and Foot and Ankle Orthopaedic Score, which were used by the developing authors, because the VISA-A is a score for patients with Achilles tendinopathy not rupture, and the Foot and Ankle Orthopaedic score has little validation data in relation to Achilles tendon ruptures [14]

### Sample size

There is no agreed optimum method for determining an appropriate sample size to evaluate aspects of validity for patient reported outcome measures [15]. However 50 patients have been advocated as the minimum requirement by previous studies evaluating aspects of validity [16]. Our planned case series of 64 patients would provide sufficient power to investigate important aspects of validity for the ATRS, and allow for 20% loss to follow-up.

### ATRS analysis plan

Internal consistency was defined as the extent to which individual items of the ATRS correlate with each other [10,15]. This is an important aspect of validity because it determines if all the items within the ATRS are measuring the same construct. This construct was defined as '*symptoms and physical activity*' by the authors who developed the ATRS. Internal consistency was evaluated using Cronbach's alpha at each time point within SPSS (v.17.0). Values between 0.7 and 0.9 are regarded as satisfactory [17].

Convergent validity was defined as the extent to which the ATRS correlated with measures consistent with its theoretically derived construct. This was evaluated by correlating the ATRS with the overall DRI and EQ 5D scores, and the subdivisions of EQ-5D related to 'mobility' and 'usual activities'and the three subdivisions of the DRI (1: common basic activities of daily life, 2: more demanding daily physical activities, 3: work related and more vigorous activities). Following an assessment of data distribution using a Shapiro-wilk analysis, the Spearman's rank correlation coefficient was used within SPSS (v 17.0) at each time point. The minimum correlation coefficient was defined as being 0.7 [15].

Responsiveness was defined as the ability of the ATRS to detect clinically important changes over time [15]. For the ATRS to be responsive it needs to demonstrate a lack of 'floor and ceiling' effects, in that an individual should not be at the maximum or the minimum value for each time point. We recorded thatfloor and ceiling effects were being present if more than 15% of respondents achieved the lowest or highest possible scores [15]. This was followed by a relative efficiency calculation to analyse responsiveness of the ATRS versus the EQ5D and DRI according to Barr *et al. *[18] . Using this method a score of greater than 1 would indicate the ATRS was more responsive than the EQ 5D and DRI and a score less than 1 would indicate the ATRS to be less responsive than the EQ 5D and DRI.

## Results

### Baseline demographics

The baseline demographics of the 64 patients can be found in Table 1. The sample reflects the broad population of patients who sustain this injury. The age range was from 21-79 years and all of the patients sustained a complete rupture of the tendon. Nineteen of the 64 patients received operative management. All 64 patients completed the above three questionnaires at baseline, which was their pre-injury scores. One patient was lost to follow-up at six weeks (63 patients, 98%). A further three were lost to follow-up at the three month time point (60 patients, 94%) and a further two patients at six months (58 patients, 91%). Fifty-six patients (88%) completed the final follow-up questionnaires at nine months.

**Table 1:** Baseline demographics

Age(Years)	44 (12)
**Male/Female**	48/16
**Left/Right**	34/30
Height (cm)*	172 (10)
Weight (Kg)*	80 (17)
**Management (Op/Non-Op)**	19/45

*Data are means (SD)

### Descriptive summary

The minimum, maximum, mean and standard deviation for the ATRS, EQ 5D and DRI at each time point can be found in Table 2, in addition to the descriptive data for the separate operative and non-operative groups. This illustrates that within the ATRS score there is a wider spread of scores at each time point, in comparison to the DRI and EQ 5D scores, which will be further discussed in the final conclusions.

**Table 2:** Overall ATRS, DRI and EQ-5D scores at each time point

Time Point		ATRS: All Patients				DRI: All Patients				EQ-5D: All Patients			
	n	Min	Max	Mean	SD	Min	Max	Mean	SD	Min	Max	Mean	SD
**Pre-Injury**	64	7	100	91	21	0	65	6	13	0.64	1.0	0.96	0.09
**6 Weeks**	63	5	88	35	19	0	81	41	17	0.12	1.0	0.7	0.19
**3 Months**	60	2	71	40	16	0	79	33	15	0.52	1.0	0.76	0.11
**6 Months**	58	15	100	66	23	0	72	16	14	0.43	1.0	0.87	0.14
**9 Months**	56	25	100	79	20	0	64	10	12	0.69	1.0	0.93	0.11
	**n**	**ATRS: Non-Operative**				**DRI: Non-Operative**				**EQ-5D: Non-Operative**			
**Pre-Injury**	45	7	100	91	23	0	65	5	14	0.64	1.0	1	0.10
**6 Weeks**	44	5	78	33	17	0	81	43	18	0.28	1.0	0.70	0.2
**3 Months**	42	2	71	40	16	0	79	33	16	0.52	1.0	0.70	0.1
**6 Months**	40	15	100	65	23	0	72	17	16	0.43	1.0	0.90	0.20
**9 Months**	38	25	100	78	20	0	64	12	13	0.69	1.0	0.90	0.10
	**n**	**ATRS: Operative**				**DRI: Operative**				**EQ-5D: Operative**			
**Pre-Injury**	19	31	100	91	18	0	31	8	11	0.73	1.0	0.9	0.10
**6 Weeks**	19	8	88	40	21	19	65	36	11	0.12	1.0	0.7	0.2
**3 Months**	18	20	69	40	14	10	48	32	12	0.56	1.0	0.8	0.1
**6 Months**	18	25	100	68	24	0	32	13	11	0.69	1.0	0.9	0.1
**9 Months**	18	39	100	81	20	0	26	7	7	0.69	1.0	0.9	0.10

### Internal consistency

The internal consistency for the ten items of the ATRS at each time point was high (Cronbach's alpha > 0.7). For pre-injury scores this was 0.98, with a decreasing score of 0.89 at six weeks and three months and increasing to 0.95 and 0.94 at the six and nine month time points (Table 3)

**Table 3:** Cronbach's alpha for the ATRS at each time point

Time Point	Cronbach's alpha
**Pre-Injury**	0.98
**6 Weeks**	0.89
**3 Months**	0.89
**6 Months**	0.95
**9 Months**	0.94

### Convergent validity

There were statistically significant (< 0.001) correlations between the ATRS and DRI scores at each time point, with correlation coefficients ranging from -0.5 to -0.9, with the exception of the nine month time point, as shown in Table 4. Table 4 also shows the 95% confidence intervals for each time point.,

**Table 4:** Convergent validity

Time Point		EQ-5D Mobility Sub-Division	EQ-5D Usual Activity Sub-Division	EQ-5D	DRI	DRI (1)	DRI (2)	DRI (3)
**Pre-Injury**	Correlation coefficient	-0.4 (-0.60 to -0.06)	-0.4 (-0.60 to -0.22)	0.4 (0.65 to 0.20)	-0.6 (-0.78 to -0.30)	-0.5 (-0.74 to -0.25)	-0.5 (-0.71 to -0.21)	-0.6 (-0.80 to -0.40)
	Significance	0.004	< 0.001	< 0.001	< 0.001	< 0.001	< 0.001	< 0.001
**6 Week**	Correlation coefficient	-0.5 (-0.64 to -0.23)	-0.3 (-0.51 to -0.07)	0.5 (0.69 to 0.33)	-0.5 (-0.68 to -0.28)	-0.5 (-0.68 to -0.33)	-0.3 (-0.50 to -0.04)	-0.3 (-0.59 to -0.13)
	Significance	< 0.001	0.019	< 0.001	< 0.001	< 0.001	0.010	0.010
**3 Month**	Correlation coefficient	-0.5 (-0.74 to -0.35)	-0.2 (-0.49 to 0.44)	0.6 (0.75 to 0.32)	-0.5 (-0.76 to -0.37)	-0.5 (-0.74 to -0.28)	-0.5 (-0.72 to -0.24)	-0.3 (-0.55 to 0.2)
	Significance	< 0.001	0.100	< 0.001	< 0.001	< 0.001	< 0.001	0.020
**6 Month**	Correlation coefficient	-0.5 (-0.70 to -0.35)	-0.6 (-0.71 to -0.26)	0.5 (0.70 to 0.21)	-0.7 (-0.80 to -0.36)	-0.5 (-0.73 to -0.24)	-0.7 (-0.85 to -0.45)	-0.6 (-0.78 to -0.41)
	Significance	< 0.001	< 0.001	< 0.001	< 0.001	< 0.001	< 0.001	< 0.001
**9 Month**	Correlation coefficient	-0.4 (-0.61 to -0.24)	-0.5 (-0.65 to -0.18)	0.3 (0.51 to 0.01)	-0.9 (-0.94 to -0.83)	-0.4 (-0.64 to -0.4)	-0.9 (-0.92 to -0.8)	-0.8 (-0.90 to -0.68)
	Significance	0.001	< 0.001	0.020	< 0.001	0.010	< 0.001	< 0.001

Data are Spearman Rank correlation coefficient (95% Confidence Intervals)

Table 4 illustrates the correlation coefficients for each construct of the DRI against the ATRS and overall EQ 5D and its two subdivisions against the ATRS at each time point. In relation to the overall EQ 5D score and its two sub-divisions the expected size of the correlations did not reach the 0.7 value, pre-defined in the analysis section. Regarding the three DRI sub-divisions, this was also not met by the first sub-division of 'common basic activities of daily life'. This criteria was only met within the last time point of the second and third sub-divisions of 'more demanding daily physical activities' and 'work related and more vigorous activities'.

### Responsiveness

Table 5 illustrates the percentage of reported responses at the top (ceiling) of the total possible scores for the ATRS, DRI and EQ 5D and the percentage of reported responses at the bottom (floor) of the possible score for the ATRS, DRI and EQ5D. All three scores demonstrated a ceiling effect (defined as > 15% respondents) for reported pre-injury scores, which was highest for the more generic quality of life measure, EQ 5D. The ATRS and DRI scores did not demonstrate a ceiling effect at any other time point. This was in contrast to the EQ 5D, which demonstrated further ceiling effects at the six and nine moth time points. None of the three outcome measures demonstrated any floor effects.

**Table 5:** Percentage of ATRS, EQ-5D and DRI respondents at either the floor or ceiling of the score

Time Point	% Ceiling			% Floor		
	**ATRS**	**EQ-5D**	**DRI**	**ATRS**	**EQ-5D**	**DRI**

**Pre-Injury**	58%	81%	47%	0%	0%	0%
**6 Weeks**	0%	6%	3%	0%	0%	0%
**3 Months**	0%	8%	0%	0%	0%	0%
**6 Months**	4%	58%	9%	0%	0%	0%
**9 Months**	11%	66%	14%	0%	0%	0%

Table 6 shows the relative efficiency of the ATRS in relation to the EQ 5D and DRI at each time point. On all occasions the ATRS demonstrated greater responsiveness when compared to the DRI and EQ 5D. At the six month time point the ATRS was 2.1 times more responsive EQ 5D and at nine months it was four times more responsive. The same trends were evident when compared to the DRI, but to a lesser extent, with the ATRS being 1.3 times more responsive at six months and 2.7 times more responsive at nine months.

**Table 6:** Relative efficiency of the ATRS across all time points

Time Point	ATRS: Z statistic derived from Wilcoxon sign rank test	EQ-5D: Z statistic derived from Wilcoxon sign rank test	DRI: Z statistic derived from Wilcoxon sign rank test	Relative Efficiency ATRS versus EQ-5D	Relative Efficiency ATRS versus DRI
**Pre-Injury/6 Week**	-6.8	-6.5	-6.7	(-6.8/-6.5)^2 ^= 1.1	(-6.8/-6.7)^2 ^= 1.0
**Pre Injury/3 Month**	-6.7	-6.3	-6.2	(-6.7/-6.3)^2 ^= 1.1	(-6.7/-6.2)^2 ^= 1.2
**Pre Injury/6 Month**	-5.5	-4.3	-4.8	(-5.5/-4.3)^2 ^= 2.1	(-5.5/-4.8)^2 ^= 1.3
**Pre Injury/9 Month**	-4.6	-2.3	-2.8	(-4.6/-2.3)^2 ^= 4	(-4.6/-2.8)^2 ^= 2.7

## Discussion

The ATRS was published in 2007, and advocated by the authors as the only validated PROM available to evaluate patients following a rupture of their Achilles tendon [10]. There have been no subsequent validation studies. Therefore, this study represents the first paper to investigate aspects of validity, outside of the developing centre.

We investigated aspects of internal consistency, convergent validity and responsiveness of the ATRS, using a sample of 64 patients. The ATRS was found to have high internal consistency at each time point (Cronbach's alpha between 0.89 and 0.95). This finding is also consistent with the original development article, which reported a Cronbach's alpha of 0.96. However, a result above 0.90 has been debated within the literature as being an indication that the outcome is too homogeneous [19,20]. The implication being that further item reduction may be appropriate [1].

Convergent validity of the ATRS was evaluated against the DRI score. Correlation coefficients between the DRI and ATRS demonstrated statistically significant correlations between the two scores at each time point. However the confidence intervals around this were wide, with only the six and nine month time points demonstrating a correlation coefficient of at least 0.7. These wide confidence intervals may be the result of the limited sample size of this study. Alternatively, they may reflect an element of heterogeneity amongst the sample. For example the inclusion of patients managed both operatively and non-operatively and patients with co-morbidities such as asthma or diabetes, may affect the distribution of PROMs scores.

These results do however provide some evidence that the ATRS is measuring similar aspects of outcome when compared to the DRI. The main limitation with this methodology was highlighted by the developing authors of the score, who acknowledged that this element of validity should be interpreted with caution as there was no existing gold-standard PROM with which to compare.

Further exploring correlations of the ATRS with the DRI we next investigated if the ATRS correlated more strongly with aspects of the three DRI sub-divisions. To further analyse aspects of convergent validity the ATRS was also correlated against the EQ-5D and two of its subdivisions evaluating 'mobility' and 'usual activities'. Again the confidence intervals were large across the time points and scales evaluated. There size of the correlations did not fulfil the pre-defined criteria of 0.7 within the EQ-5D or its subdivisions. Within the DRI this criteria was met by the second and third sub-divisions of the DRI at the six and nine month time points. These results were anticipated by the authors to an extent because the EQ-5D measures more generic quality of life, as opposed to the alternative construct of physical activity, measured by the ATRS. Again the key limitation of these correlations is that the three scores are measuring only similar constructs as opposed to exact constructs and with the large confidence intervals reported, a larger sample may be required. A 'foot and ankle' specific PROM may provide a more exact construct for comparison, but as described in the methods section there is also a distinct lack of robustly-developed outcome measures in this area [14].

The more specific ATRS outcome measure demonstrated greater responsiveness than the more generic DRI and EQ-5D scores at each time point. These results were in keeping with the original development article. The level of responsiveness was only marginal in comparison to the DRI and EQ-5D up until the three month time point, with greater levels of responsiveness evident at the six month and nine month time points. This may by representative of the greater ceiling effects seen within the EQ-5D and DRI scores. There are many methods available to determine responsiveness. This method was used as opposed to more routinely reported effect sizes because it does not require parametric assumptions.

## Conclusion

This study provides further evidence regarding the validity of a newly developed measurement tool. Overall the ATRS demonstrated high internal consistency and responsiveness in comparison to the EQ-5D and DRI. It has also demonstrated minimal ceiling effects (within pre-injury scores only), and no floor effects. There was limited evidence of convergent validity with the DRI and EQ5D, although these tools do measure slightly different constructs. The descriptive data in Table 2 will provide researchers with a useful resource of estimates of range and spread of scores across multiple time points, but further research will be required to determine minimally important differences within this score.

Future areas of research could explore the use of this tool in the separate operative and non-operative populations, and within patients with chronic rupture presentations. While there is certainly scope to further explore aspects of validity of this new score, ideally with even larger samples, this study is a positive step towards the use of a universal measure of outcome for patients with a rupture of the Achilles tendon.

## Abbreviations

DASH: Disabilities of the arm shoulder and hand score; ATRS: Achilles tendon total rupture score; DRI: Disability rating index; PROM: Patient reported outcome measures.

## Competing interests

This trial was funded through Arthritis Research UK. The authors declare that they have no competing interests.

## Authors' contributions

All authors have made substantial contributions to conception and design of this study. RK contributed to acquisition of data, RK, NP, SL and MC contributed to the analysis and interpretation of data. RK and MC have been involved in drafting the manuscript and NP, JA and SL have contributed to revising it critically for important intellectual content. All authors have given final approval of the version to be published. Each author has participated sufficiently in the work to take public responsibility for appropriate portions of the content.
